# Novel Camelid Antibody Fragments Targeting Recombinant Nucleoprotein of *Araucaria hantavirus*: A Prototype for an Early Diagnosis of Hantavirus Pulmonary Syndrome

**DOI:** 10.1371/journal.pone.0108067

**Published:** 2014-09-22

**Authors:** Soraya S. Pereira, Leandro S. Moreira-Dill, Michelle S. S. Morais, Nidiane D. R. Prado, Marcos L. Barros, Andrea C. Koishi, Giovanny A. C. A. Mazarrotto, Giselle M. Gonçalves, Juliana P. Zuliani, Leonardo A. Calderon, Andreimar M. Soares, Luiz H. Pereira da Silva, Claudia N. Duarte dos Santos, Carla F. C. Fernandes, Rodrigo G. Stabeli

**Affiliations:** 1 Fundação Oswaldo Cruz, Fiocruz Rondônia, Porto Velho, RO, Brazil; 2 Instituto Carlos Chagas, Fiocruz Paraná, Curitiba, PA, Brazil; 3 Departamento de Medicina, Universidade Federal de Rondônia, UNIR, Porto Velho, RO, Brazil; 4 Centro de Pesquisa em Medicina Tropical, CEPEM, Porto Velho, RO, Brazil; Division of Clinical Research, United States of America

## Abstract

In addition to conventional antibodies, camelids produce immunoglobulins G composed exclusively of heavy chains in which the antigen binding site is formed only by single domains called VHH. Their particular characteristics make VHHs interesting tools for drug-delivery, passive immunotherapy and high-throughput diagnosis. Hantaviruses are rodent-borne viruses of the Bunyaviridae family. Two clinical forms of the infection are known. Hemorrhagic Fever with Renal Syndrome (HFRS) is present in the Old World, while Hantavirus Pulmonary Syndrome (HPS) is found on the American continent. There is no specific treatment for HPS and its diagnosis is carried out by molecular or serological techniques, using mainly monoclonal antibodies or hantavirus nucleoprotein (N) to detect IgM and IgG in patient serum. This study proposes the use of camelid VHHs to develop alternative methods for diagnosing and confirming HPS. Phage display technology was employed to obtain VHHs. After immunizing one *Lama glama* against the recombinant N protein (prNΔ_85_) of a Brazilian hantavirus strain, VHH regions were isolated to construct an immune library. VHHs were displayed fused to the M13KO7 phage coat protein III and the selection steps were performed on immobilized prNΔ_85_. After selection, eighty clones recognized specifically the N protein. These were sequenced, grouped based mainly on the CDRs, and five clones were analyzed by western blot (WB), surface plasmon resonance (SPR) device, and ELISA. Besides the ability to recognize prNΔ_85_ by WB, all selected clones showed affinity constants in the nanomolar range. Additionaly, the clone KC329705 is able to detect prNΔ_85_ in solution, as well as the native viral antigen. Findings support the hypothesis that selected VHHs could be a powerful tool in the development of rapid and accurate HPS diagnostic assays, which are essential to provide supportive care to patients and reduce the high mortality rate associated with hantavirus infections.

## Introduction

Antibody engineering has allowed for the development of many forms of antibodies for diagnostic and therapeutic use in recent decades [1]. Minimization of monoclonal antibodies to obtain monovalent antibody fragments (Fab), single chain variable fragments (scFv) and even single domains has been employed to produce antibodies that can be used in biosensors, for tumor-targeting, drug-delivery or passive immunotherapy [2], [3], [4].

In addition to conventional antibodies, camelids produce functional immunoglobulins composed only of heavy chains in which the antigen binding site is formed only by the single N-terminal variable domain, referred to as VHH [5], [6], [7]. With an approximate molecular weight of 15 kDa, VHH fragments are one-tenth the size of whole antibodies [3], [8]. Their small size, along with their ability to recognize weakly antigenic epitopes or epitopes that are inaccessible to conventional antibodies, their high solubility, thermal and pH stability, ability to cross dense tissues and lower production costs make VHHs versatile tools for biotechnological applications [3], [9], [10], [11]. Among VHH's applications is the development of medicines for the treatment of rheumatoid arthritis and neurodegenerative disorders, as well as antitumor and antiviral drugs [4], [12]–[17]. VHHs have also been applied in cell imaging studies, *in vivo* imaging of tumor tissue and to diagnose viral infections [4], [18], [19].

Hantaviruses are rodent-borne viruses that belong to the Bunyaviridae family and can cause Hemorrhagic Fever with Renal Syndrome (HFRS), more commonly found in the Old World, and Hantavirus Pulmonary Syndrome (HPS), present mostly in the American continent [20], [21]. Since 1993, about 617 cases of HPS were reported in the United States [22]. Outside of North America, clusters of HPS cases have been reported in Argentina, Bolivia, Chile, Ecuador, Paraguay, Panama, Uruguay, Venezuela, and Brazil, where 1634 cases have been recorded [23].

Hantavirus infections have a high case-fatality rate (between 35 to 50%), and no specific treatments are available. Therefore, accurate and rapid diagnosis early in the disease course is essential to assure supportive care and decrease mortality in infected patients [24].

Current diagnostic methods for HPS include molecular and serological assays [24], [25]. Traditionally, ELISA methods aiming to improve the specificity and sensitivity of hantavirus detection have been developed using mainly the recombinant nucleoprotein to detect IgM and IgG in the patients' serum [24], [25], [26]. Monoclonal antibodies directed to the recombinant nucleoprotein were reported to enhance the diagnosis rate of HPS [27]. Nucleoprotein (N protein), the most antigenic hantavirus protein, is detectable early in the infection course and participates in the viral replication [28], [29], [30], [31], [32]. Besides encoding the N protein, the hantavirus genome encodes the viral RNA polymerase and a glycoprotein precursor (GPC), which is cleaved into two surface glycoproteins G1 and G2 that seem to be associated with the viral infection process [33].

Given the properties presented by camelid VHHs, the characteristics of the hantavirus nucleoprotein and the necessity of developing high-throughput, accurate, rapid, reliable, low-cost diagnostic methods for hantavirus infections, as well as the need for alternative approaches to prevent or control the course of the disease, our study aimed to identify VHHs capable of recognizing the prNΔ_85_ of *Araucaria hantavirus*. Anti-prNΔ_85_ VHHs selected through the phage display library, generated from an immunized *Lama glama*, specifically recognized the hantavirus antigen by ELISA, western blot, and also in sensor devices utilizing a surface plasmon resonance set-up.

## Material and Methods

### Ethics statement

All experimental procedures involving animals were carried out in accordance with the recommendations of the National Council for the Control of Animal Experimentation (CONCEA), and were approved by the institutional Ethics Committee on Animal Use (CEUA) under protocol 2012/11.

### Antigen, strains and other reagents

Recombinant nucleoprotein ARAUV prNΔ_85_ [24] was obtained from Instituto Carlos Chagas/Fiocruz Paraná. Home-made anti-IgG_2,3_ was produced by immunizing rabbits with previously isolated *Lama glama* IgG_2_/IgG_3_. Peroxidase conjugated Mouse anti-rabbit IgG was purchased from Sigma Aldrich. *E.coli* TG1 (Stratagene, La Jolla, USA) and HB2151 (provided by Dr. Gerhard Wunderlich, USP, Brazil) strains were used, respectively, for the cloning of VHH and the expression of the selected clones. The pHEN1 phagemid provided by Dr. Pierre Lafaye (Institute Pasteur, Paris, France) was used to clone and produce the VHH immune library after the insertion of the 6xHis-tag sequence by site-directed mutagenesis PCR (pHEN1-6xHis), and the M13KO7 helper phage purchased from New England Biolabs (Ipswich, USA) was used to produce the secondary immune library. Mouse monoclonal antibody (Mab 432/6BF) is an IgG1κ antibody against the nucleoprotein (rNΔ_85_) of the Araucaria hantavirus strain (ARAUV) [27]. Rodent serum was collected during fieldwork of epidemiology surveillance for hantavirus in General Carneiro, Paraná, Brazil in 2006 and 2010. These samples were previously tested for the presence of hantavirus IgG antibodies, and when positive, sequencing was performed to identify the virus genotype [34].

### Purification of *Lama glama* IgG and rabbit anti-IgG_2, 3_ preparation

Approximately 8.5 mL of *Lama glama* serum was centrifuged at 15,000 xg for 15 minutes to remove the remaining particles from blood extraction. After that, the supernatant was recovered in an Erlenmeyer flask (50 mL) for Ammonium Sulphate precipitation [35]. Subsequently, the precipitate was dissolved in 3 mL of 50 mM Tris, containing 150 mM NaCl, pH 8.1, and applied to a Sepharose-G column (3 mL) equilibrated in the same buffer. The chromatography was performed in the same buffer until the column was washed with 20 x of column volume at a flux of 2 mL/min. The IgG fraction was eluted with 6 mL of 80 mM Glycine, pH 2.5, containing 250 mM NaCl. Samples were recovered, dialyzed against deionized water and concentred using a 5 mL Amicon ultra filter. The concentrated sample was applied to a 12% SDS-PAGE gel in its own well. After electrophoresis, the bands corresponding to IgG_2_ and IgG_3_ were cut out from the gel and briefly washed in deionized water. The gel was sliced, washed in a small amount of 50 mM PBS pH 7.8 and homogenized by passing it through a large gauge needle. The samples were divided into three equal parts and were used for rabbit immunization [36]. After immunization, 40 mL of blood was collected and prepared for serum production. About 8 mL of serum was collected and the IgG purification was performed in accordance with the method above. Rabbit IgG anti-IgG_2, 3_ was titled among IgG_2, 3_ previously purified utilizing ELISA methods. The determined title for use was 1∶12000. Thus, the rabbit IgG anti-IgG_2, 3_ was aliquoted, lyophilized and stored for use.

### Animal immunization

One young adult male *Lama glama*, with food and water available *ad libitum*, was immunized at fortnightly intervals with a mixture of 200 µg of prNΔ_85_ and 200 µL of complete or incomplete Freund's adjuvant (Sigma-Aldrich, Saint Louis, USA) via subcutaneous injections. A third immunization was carried out intravenously without Freund's adjuvant in 500 µL of 0.9% NaCl. Blood was obtained 7 days later for lymphocyte isolation (Table 1). During the immunization protocol, the immune response was monitored through ELISA.

**Table 1:** Llama immunization schedule.

DAY	IMMUNIZATION NUMBER	SERUM COLLECTION	PROTEIN CONCENTRATION
0	1	Yes	200µg associated with complete Freund's adjuvant
7	–	Yes	
14	2	Yes	200µg associated with incomplete Freund's adjuvante
21	–	Yes	
28	3	Yes	200µg in 500 µL 0.9% NaCl
35	–	Yes	

The *Lama glama* was immunized with the recombinant nucleoprotein ARAUV, strain HPR 02-72. The blood was collected from the jugular vein seven days after the third immunization.

### Immune llama VHH library construction

Following the isolation of peripheral blood lymphocytes using Ficoll-Paque Plus (GE Healthcare, Little Chalfont, United Kingdom), total RNA extraction was performed using Trizol Reagent (Invitrogen, Carlsbad, USA), and cDNA synthesis was carried out using the SuperScript III First-Strand Synthesis System for RT-PCR (Invitrogen) according to manufacturer's instructions. The VHH repertoire was amplified with two pairs of genespecific primers (VH BACK A6: 5′ GAT GTGCAGCTGCAGGCCTCTGG(A/G)GGAGG 3′ and CH2 FOR TA4: 5′ CGCCAT CAAGGTACCAGTTGA 3′, and VHFOR36: 5′ ATGCCATGACTGCGGGCCCAGCCGGCCATGGCCGA(G/C)GT(G/C)CAGCT 3′ and VH BACK A4: 5′ GGACTAGTTGCGGCCGCTGAGGAGACGGTGAC GGTGACCTG 3′) in two consecutives reactions of RT-PCR (recognition sites for *Sfi* and *Not*I are underlined). The first fragment, with about 600 bp, corresponds to the VH-CH2 immunoglobulin G region, whereas the second product, with 400 bp, is related to the VHH fragment of camelid immunoglobulins [37]. The obtained RT-PCR products were purified, restriction digested, and inserted into *Sfi* and *Not*I sites of the pHEN1-6xHis phagemid vector, in frame with the M13 gene III for expression of VHH-6xHis-PIII fusion protein. Ligation reactions were transformed into home-made electrocompetent *E. coli* TG1 in order to obtain the VHH primary library. After construction, the library size was determined by plating the transformation product on 2YT/ampicillin/glucose (amp/glu) agar plates.

Recombinant phages expressing anti-prNΔ_85_ VHHs, conjugated to the minor coat protein g3p, were produced following the infection of VHH library with helper phage M13KO7. *E. coli* TG1 cells grown in 2YT/amp/glu medium at 37°C were inoculated in log-phase with M13KO7 helper phages and incubated at 37°C for 1 h without shaking and 1 h under shaking. Afterwards, the bacterial culture was centrifuged at 3000 rpm for 15 min, the medium was replaced by 2YT containing 100 µg/mL ampicillin and 25 µg/mL kanamycin, and the culture was incubated overnight under shaking at 30°C. Subsequently, the material was centrifuged, and the supernatant was PEG precipitated (20% polyethylene glycol 6000 in 2.5 M NaCl in water) at 4°C for 1 h. Phages were spun-down, the pellet resuspended in 1 mL PBS, titrated and stored at −20°C.

### Panning for anti-prNΔ_85_ VHHs

To select anti-prNΔ_85_ VHHs, immunotubes MaxiSorb (Sigma-Aldrich, Saint Louis, USA) were adsorbed with 300 µg of prNΔ_85_ in 3 mL of 1X PBS pH 7.4 overnight at 4°C. After washing three times with PBS, the reactions were blocked with blocking solution - BS (5% skimmed milk in PBS) for 1 h. Then, phages expressing the VHH repertoire, previously incubated for 30 min at 37°C in BS, were added to the immunotubes and the samples were incubated for 1 h with shaking and 1 h without shaking at 37°C. The immunotubes were washed five times with PBST (1XPBS and 0.05% Tween-20), and five more times with 1X PBS. Next, the phage elution was washed with 1 mL of 100 mM HCl and neutralized with 1 mL of 1 M Tris-HCl. Then, eluates were transferred to *E. coli* TG1 (A_600_ 0.5), and the cultures were incubated for 30 min without shaking and 30 min with shaking at 37°C. After centrifugation at 4000 rpm for 15 min, the supernatant was discarded, the pellet resuspended in 500 µL of 2YT, plated on 2YT/amp/glu, and incubated overnight at 30°C. Colonies were selected to perform colony PCR and ELISA analysis, and plates were scraped to carry out the next round of panning or stored at −80°C.

### Screening ELISA

To verify the specificity of each clone, soluble VHHs were expressed in 2 mL tubes containing 2TY/amp plus 1 mM IPTG for 16 h at 30°C. After centrifugation, the supernatants were used for ELISA assays. For this, microtiter plates (*Immuno 96 MicroWell Plates*, Nunc Maxisorp, Sigma-Aldrich) were coated with 1 µg of prNΔ_85_ or yellow fever attenuated virus/well and incubated overnight at 4°C. Wells were washed with PBST, unspecific sites blocked with BS and 50 µL of culture supernatant containing soluble VHHs were added to the wells and incubated for 1 h. Excess VHHs were removed by washing the samples five times with 300 µL PBST, and the rabbit IgG anti-IgG_2, 3_ at a 1∶12000 dilution for 16 h in BS was added. Wells were washed with PBST 5 times, and peroxidase conjugated mouse anti-rabbit IgG antibody was incubated at a 1∶40000 dilution for 2 h in BS. TMB system solution and 100 mM HCl were used to reveal and stop the reaction, respectively. The absorbances were measured at 450 nm in a microplate reader (*BioTek-Synergy HT*, Bio-Tek, Highland Park, USA). Positive clones were sequenced, analyzed and deposited into GenBank. As a positive control, llama hantavirus immune serum was used. While the negative control was performed using the llama pre-immune serum.

### Expression and purification of anti-prNΔ_85_ VHH

After sequencing, five clones (Genbank accession no. KC329704–KC329708) were selected and subcloned into the nonsuppressor *E. coli* strain HB2151. Colonies with apparently intact VHH fragments (checked by PCR, results not shown) were grown in 250 mL 2YT broth containing 100 µg/mL ampicillin under shaking conditions at 37°C, and when an OD_600_ of 0.9 was reached, 1 mM IPTG (Promega, Madison, USA) was added to induce VHH expression. The cultures were grown overnight at 30°C and were centrifuged at 2700 rpm for 15 min.

Bacterial lysates were obtained by resuspending the individual bacterial pellets in 10 mL of 50 mM Tris-HCl pH 8.0, incubating the samples with 100 mg/mL of lysozyme for 20 min at room temperature, performing the sonication for 3.5 min, with 1 min pulses (Misonix Ultrasonic Processor, Qsonica, Newtown, USA), and then centrifuging them at 8000 rpm for 15 min. 6xHis-tagged VHHs were purified using Ni-NTA Agarose resin (Qiagen, Hilden, Germany) according to manufacturer's instructions. After Ni-NTA metal-affinity chromatography, the eluates were concentrated using Amicon Ultra-2 filter devices (Merck Millipore, Billerica, USA) and protein concentration was determined using the Bradford test.

### SDS PAGE and Western blot analysis

Expression and purification were verified through a 12% sodium dodecylsulphate polyacrylamide gel electrophoresis (SDS-PAGE). The gel was stained with colloidal Coomassie brilliant blue R-250 (Dinâmica, Diadema, Brazil). For Western blot analysis, approximately 12 µg prNΔ_85_ was reduced in sample buffer and electrophoresed on a 10% SDS-PAGE. Following the electrophoresis, proteins were transferred to a nitrocellulose membrane (Amersham LifeScience, Little Chalfont, United Kingdom), and reactive sites were blocked with TBSM (5% skimmed milk in 1X TBS) at room temperature for 18 h on a shaker. After washing, the membrane was sliced and the strips incubated overnight with 2 µg/mL of purified anti-prNΔ_85_ monoclonal antibody (Mab432/6BF) [24], 2 µg/mL of purified anti-prNΔ_85_VHHs, 2 µg/mL of unrelated VHH (anti-BthTX-I, Bothrops toxin I, data not published), and llama pre-immune serum, in TBSM. Membranes were washed three times for five min in TBST (0.1% Tween 20+1XTBS) and incubated with home-made anti-IgG_2, 3_ produced in rabbit (1∶10000 in 5% TBSM). To the strip previously incubated with anti-prNΔ_85_ monoclonal antibody, an HRP-conjugated anti-IgG produced in mouse (Sigma) in a 1∶10000 dilution was applied. After overnight incubation, the strips were washed with 1X TBST and incubated with DAB solution (30% H_2_O_2_, 1 M Tris-HCl and 0.028 g DAB) for 10 min for signal detection. The ColorPlus Prestained Protein Marker (New England BioLabs) was used as a molecular weight standard.

### Affinity measurement by Surface Plasmon Resonance (SPR)

Studies on the interaction between prNΔ_85_ and KC329704, KC329705, KC329706, KC329707, KC329708 anti-prNΔ_85_VHHs were performed by SPR spectroscopy using a Biacore T200 system (GE Healthcare). The prNΔ_85_ was immobilized on carboxymethylated dextran CM5 chips by amine coupling [38]. The dextran layer of the sensor chip was activated by a 1∶1 (v/v) mixture of 0.4 M EDC (1-Ethyl-3-(3-dimethylaminopropyl)carbodiimide) and 0.1 M NHS (N-hidroxysuccinimide) at a flow rate of 5 µL/min. The prNΔ_85_ was diluted in 10 mM acetate buffer (pH 5.5) at a concentration of 50 µg/mL and then injected into a selected flow cell until a surface of 487.4 resonance units (RUs) was obtained. A second flow cell was used as blank control. After immobilization, a solution of 1 M ethanolamine hydrochloride was injected in order to block remaining reactive groups in both flow cells. For kinetic measurements, stock solutions of purified anti-prNΔ_85_VHHs were diluted with the assay running buffer (0.1 M phosphate buffer, 27 mM KCl and 1.37 M NaCl, pH 7.4) in order to prepare a concentration series from 50 to 0.78 µg/mL). The samples were then infused into two serially connected flow channels (Fcs) at a flow rate of 30 µL/min at 37°C. Chip regeneration was done with AIW (1∶1∶1) followed by CIW (1∶1∶1) solutions for 30 seconds each [A - Equal volumes of oxalic acid, H_3_PO_4_, formic acid, and malonic acid, each at 0.15 M, adjusted to pH 5.0 with 4 M NaOH; C - 20 mM EDTA; I - KSCN (0.46 M), MgCl_2_ (1.83 M), urea (0.92 M), guanidine-HCl (1.83 M); W - deionized water] [39]. The binding responses were obtained by subtracting the RUs obtained from the blank control cell and assay running buffer-only injections. Kinetic analysis was performed by fitting the obtained sensogram with a 1∶1 Langmuir model using the BIA-evaluation software (GE Healthcare Life Sciences, USA).

### Detection of soluble prNΔ_85_ and native viral antigen

For detection of soluble prNΔ_85_, as well as viral antigens in serum samples of naturally infected mice, KC329704, KC329705, and KC329706 anti-prNΔ_85_VHHs, or KC329705 anti-prNΔ_85_VHH (500 ng/well), respectively, were adsorbed to a solid surface in 96 microwell plates (Nunc Maxisorp) for 18 h at 4°C. After this incubation step, the plates were blocked with 2% skim milk, 0.05% Tween-20 PBS (blocking buffer) for 30 minutes at 37°C, and washed three times with washing buffer (0.01% Tween-20 PBS). prNΔ_85_ (0, 100 and 200 ng/well) or rodent serum, diluted 1∶100 in blocking buffer, were added to the plates and incubated for 1 hour at 37°C. Then the plates were washed three times and the monoclonal antibody 432/6BF (pure hybridoma culture supernatant) was added for another hour at 37°C. Finally, the plates were washed three times, the goat anti-mouse IgG peroxidase conjugated incubated for 1 hour at 37°C, and TMB substrate (KPL, Gaithersburg, USA) was added and incubated for 15 min to allow for color development. The reaction was stopped with 2N H_2_SO_4_. Absorbance was read at a wavelength of 450 nm in a microplate reader (Synergy H1M, Biotek, USA). During the detection of native viral antigen, the hantavirus recombinant nucleoprotein (200 ng/well) was used as a positive control. To perform the negative control no anti-prNΔ_85_VHHs were added to the plates.

Additionally, tissues (lung, liver, and/or kidney) from antibody-positive rodents were analyzed by RT-PCR to amplify the partial S segment. The molecular tests were carried out using various sets of primers [34]. PCRs with the partial S genome segment (434 nucleotides) of the N-encoding region were conducted using an nRT-PCR with primers designed to detect hantaviruses associated with sigmodontine rodents. The specific primers were designed based on the partial N sequence and used for the nRT/PCR. cDNA was synthesized using primer F166–189, with Superscript II Reverse Transcriptase (Invitrogen Inc, Carlsbad, CA). Five microliters of cDNA was then amplified by PCR. Two PCR cycles were performed with the primers F166–189 (5′-AGCACATTACAAAGCAGACGGGCA-3′) and R1054-1071 (5′-AGCCATGATTGTGTTGCG-3′) for the first PCR cycle and F274–291 (5′-CCAGTTGATCCAACAGGG-3′) and R664–690 (5′-TATGATATTCCTTGCCTTCACTTGGGC-3′) for the second PCR cycle. This yielded a fragment of 416 base pairs. The RT step (1 hour at 42°C) was followed by thermal cycling (95°C for 2 minutes, then 40 cycles at 95°C for 30 seconds, 38°C for 30 seconds, and 72°C for 2 minutes). The thermal cycling conditions were similar for the nested PCR, except that a higher annealing temperature (42°C) was used. Reactions were then incubated at 72°C for 10 minutes for a final extension cycle. The PCR products were subjected to electrophoresis on 1% agarose gels, stained with ethidium bromide [40].

## Results

### Humoral response and generation of recombinant anti-prNΔ_85_ VHHs

**Figure 1:**
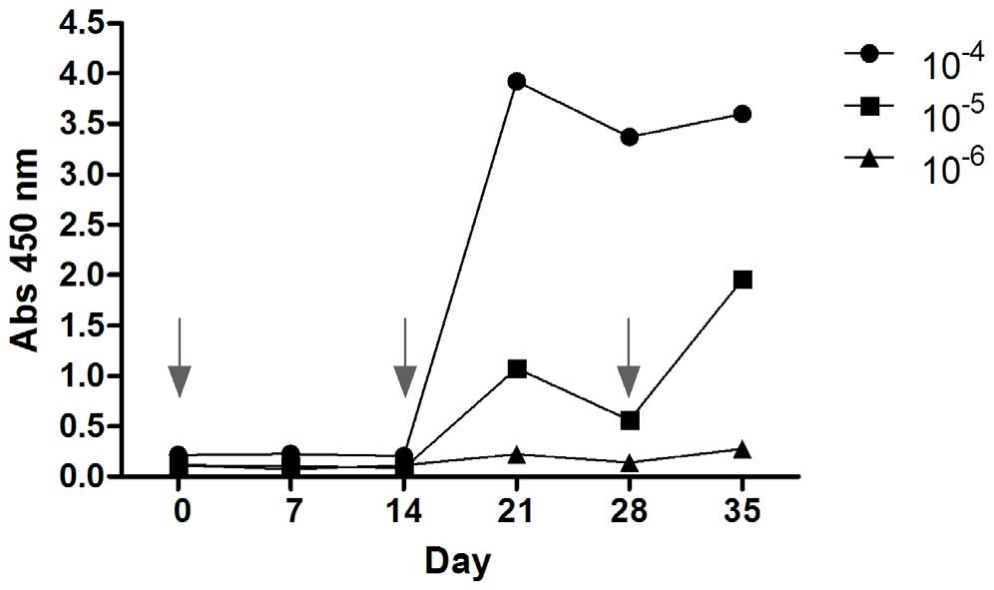
Monitoring the llama immune response by ELISA. The animal showed a rapid and strong response against the prNΔ85 protein after the second immunization (i.e., by Day 21; see immunization schedule Table 1). A sharp decrease in the humoral response was observed at day 28, when the third immunization was carried out. The final bleed performed on day 35, showed an antiserum titre of 1.0×10^−6^. The arrows indicate the immunization days.

The humoral response to prNHΔ_85_ was monitored over the course of the *Lama glama* immunization schedule. Using 0.2 mg prNΔ_85_ the animal did not suffer any visible signs of inflammation or local reaction at the injection sites. The llama elicited a rapid response to the recombinant protein after administering two injections (post-immune day 21). Subsequently, a sharp decrease in the humoral response to prNHΔ_85_ was observed on day 28. After the third immunization, the antiserum titre, assessed by indirect ELISA, increased considerably (day 35). The serum dilution that corresponded to 3x the value of the pre-immune sera was determined as 1×10^6^ (Figure 1).

To generate recombinant anti-prNΔ_85_ VHHs, the phage display method was employed. After immunizing the llama with the hantavirus nucleocapsid protein and monitoring the animal immune response through ELISA, VHH regions were amplified by RT-PCR using cDNA synthesized from total RNA extracted from about 6.8×10^6^ peripheral lymphocytes. These regions were then cloned into the phagemid pHEN1-6xHis to construct an immune VHH library with 3×10^7^ clones. A total of 1.7×10^11^ cfu/mL VHHs displaying phage particles were obtained after co-infecting the phagemid transfected TG1 *E. coli* strain with the M13K07 helper phage in the phage rescuing strategy. Subsequently, the generated phage antibody immune library was subjected to two selection rounds, performed on immobilized prNΔ_85_ protein, to identify VHH clones that recognize specifically the recombinant hantavirus protein. Colony PCR was performed in order to verify the presence of VHH in twenty-six and ninety-two clones, obtained after the first and second rounds, respectively (data not shown).

### Specificity of selected clones

Once confirmed by colony PCR, one hundred and eighteen VHH clones were expressed in *E. coli* TG1 to verify the clone reactivity by ELISA. Of these, eighty clones were able to recognize the recombinant prNHΔ_85_ protein. Thirteen were positive clones derived from the first round of selection, demonstrating that one round of bioppaning was already sufficient to identify 50% positive phages. The other 67 positive selected VHHs were products of the second round (Figure S1). All clones that showed an absorbance value (OD 450 nm) higher than the stipulated cut off point (3 mean OD from negative samples plus 3 standard deviations) were considered positive. VHHs reacted with prNΔ_85_ protein, but not with yellow fever attenuated virus (Figure S2).

### Characterization of the anti-prNΔ_85_ VHHs

After plasmidial DNA extraction, positive clones were sequenced, analyzed and the 11 sequences that showed different profiles were deposited in GenBank under accession numbers KC329698, KC329699, KC329700, KC329701, KC329702, KC329703, KC329704, KC329705, KC329706, KC329707, and KC329708.

**Figure 2:**
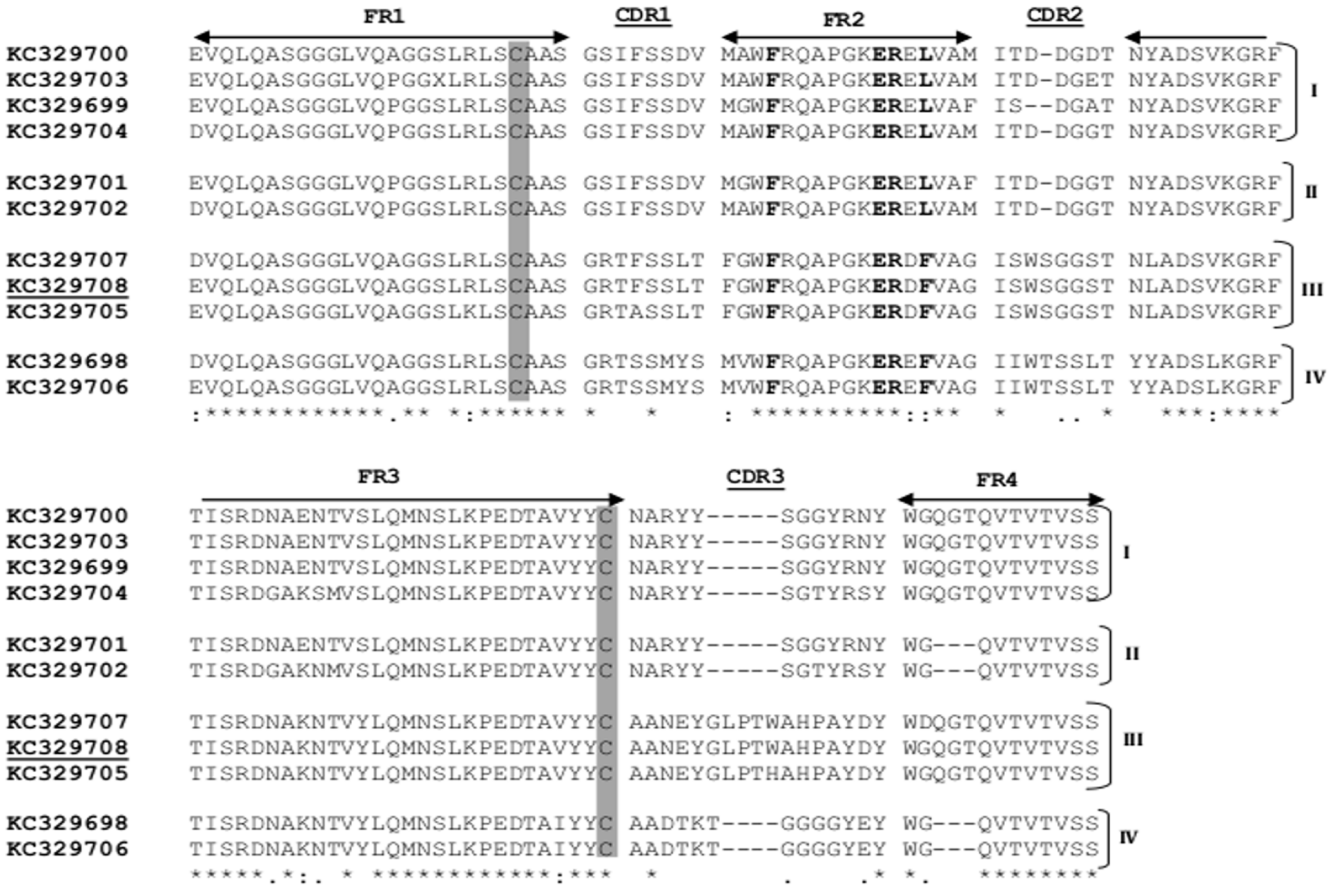
Nucleoprotein binding VHHs. Protein sequence of 11 VHH antibody fragments selected by phage display. The framework regions are indicated with arrows; three complementarity determining regions CDR1, CDR2, and CDR3 are denoted; two conserved cysteines are shaded; VHH hallmark substitutions in the FR2 are bolded; Sequence profiles are clustered in four groups (I–IV).:  =  highly conserved amino acids; *  =  identical amino acid residues;.  =  different amino acids somewhat similar; blank  =  dissimilar amino acids or gaps.

Based on amino acid similarity, the identified sequences were grouped into four clusters of VHH (I–IV) with 4, 2, 3, and 2 members, respectively (Figure 2). This grouping was proposed considering the sequence variability, mainly observed in the complementarity determining regions (CDR), CDR2 and CDR3, as well as the length of the CDR3 region. Cluster III (KC329705, KC329707, and KC329708) presented 17 amino acid residues in CDR3, while clusters I (KC329699, KC329700, KC329703, and KC329704), II (KC329701 and KC329702), and IV (KC329698 and KC329706) showed 12 or 13 amino acids in the same hypervariable region. Furthermore, two conserved cysteine residues that form the canonical cross-species disulfide bond between framework 1 (FR1) and FR3 and the well established hallmark amino acids in FR2 (Phe37, Glu44, Arg45, Leu/Phe47) were detected on all identified sequences. Aiming to verify the ability of anti-prNΔ_85_ VHHs to recognize the recombinant hantavirus protein by western blot, SPR, and ELISA, five clones (KC329704, KC329705, KC329706, KC329707, and KC329708) representing clusters I, III and IV were expressed in *E.coli* HB2151 at a one liter scale and purified by a Ni-NTA resin.

**Figure 3:**
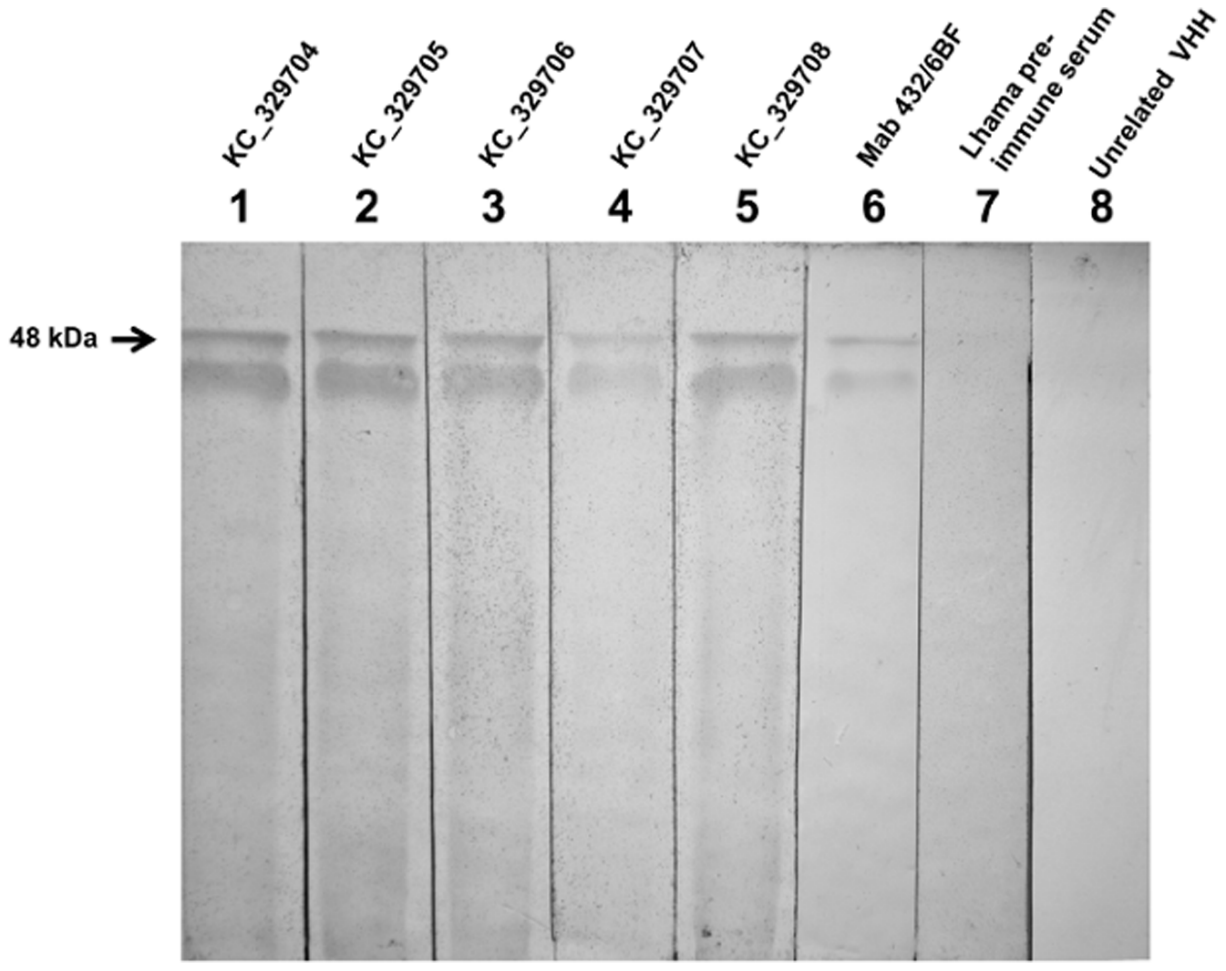
Western blot analysis of anti-prNΔ_85_ VHHs and mouse monoclonal antibody against prNΔ_85_. 10% SDS-PAGE was carried out to resolve the purified prNΔ_85_. Protein samples were labeled with anti-prNΔ_85_VHHs, (KC329704–KC329708) (lanes 1–5), mAb anti-rNΔ_85_ (lane 6), as well as the llama pre-immune serum (lane 7), and unrelated VHH (anti-BthTX-I), as negative controls (lane 8).

Western blot analysis demonstrated that all purified anti-prNΔ_85_ VHHs could bind to prNΔ_85_ (48 kDa), used to construct the llama VHH library, but were not able to react against the llama pre-immune serum, or unrelated VHH, indicating the clone's specificity. Mouse monoclonal antibody anti-prNΔ_85_ (Mab432/6BF), which specifically recognizes the recombinant hantavirus nucleoprotein [27] revealed a similar pattern in the western blot, confirming that the 48 kDa band corresponded to the recombinant hantavirus protein (Figure 3).

### Interaction Analysis by SPR

**Figure 4:**
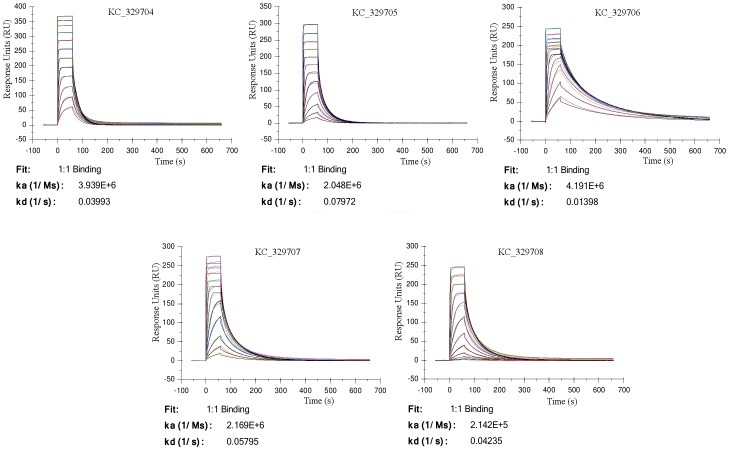
Surface plasmon resonance analysis to show specific binding of VHH and monoclonal antibody. Sensorgrams obtained after injection of purified VHHs (KC329704–329708) at concentrations of 8, 4, 2, 1, 0.5, 0.25, 0.125, 0.063, 0.0313, 0.0156, 0.00781, 0.0039 µM.

The affinity between immobilized prNΔ_85_ (ligand) and purified KC329704, KC329705, KC329706, KC329707, KC329708 anti-prNΔ_85_ VHHs (analytes) in a label-free antigen-binding assay using surface plasmon resonance (SPR) showed sensograms of binding and dissociation between the immobilized ligand and analytes at concentrations of 8 to 0.0039 µM. The obtained curves show a variation of the binding associated with the concentration of the injected anti-prNΔ_85_VHHs. The highest curve corresponds to the binding between the surface and the anti-prNΔ_85_ VHHs at 8 µM, while the smaller curve corresponds to the lowest concentration of analytes (0.0039 µM) (Figure 4). The fitting of sensogram of binding and dissociation with a 1∶1 Langmuir model performed using the Biacore software allow the evaluation of association rate constants (k_on_), dissociation rate constants (k_off_) and equilibrium dissociation constants (K_D_), corresponding to k_off_ divided by k_on_ (Table 2). All tested anti-prNΔ_85_ VHHs showed binding constant values in the nanomolar (10^−7^ to 10^−9^) range, which permit consider all high affinity antibodies. The KC329706 anti-prNΔ_85_VHH clone showed the highest affinity in the low nanomolar range (K_D_ 3.3 nM) to immobilized prNΔ_85_.

**Table 2:** Kinetic analysis and ranking of selected llama anti-prNΔ_85_ VHH clones; ranking by highest to lowest affinity.

Clone	k_on_	k_off_	K_D_	R_max_	Chi^2^
	[1/Ms]	[1/s]	[nM]	[RU]	
KC329706	4.191×10^6^	0.01398	3.3	190.3	15.4
KC329704	3.939×10^6^	0.03993	10.1	195.8	36.7
KC329707	2.169×10^6^	0.05795	26.7	205.2	11.9
KC329705	2.048×10^6^	0.07972	38.9	199.0	3.3
KC329708	2.142×10^5^	0.04235	197.7	194.6	7.6

k_on_ - association rate constant; k_off_ - dissociation rate constant; K_D_ - equilibrium dissociation constant; R_max_ - response at saturation; Chi^2^ - mean squared of the signal noise.

Additionally, the low Chi^2^ values obtained for the fitting of the sensograms to the 1∶1 Langmuir curve reflect the high accuracy of this model and the high confidence of the kinetic paramethers obtained (Figure 4).

### Detection of soluble prNΔ_85_ and native viral antigen

**Figure 5:**
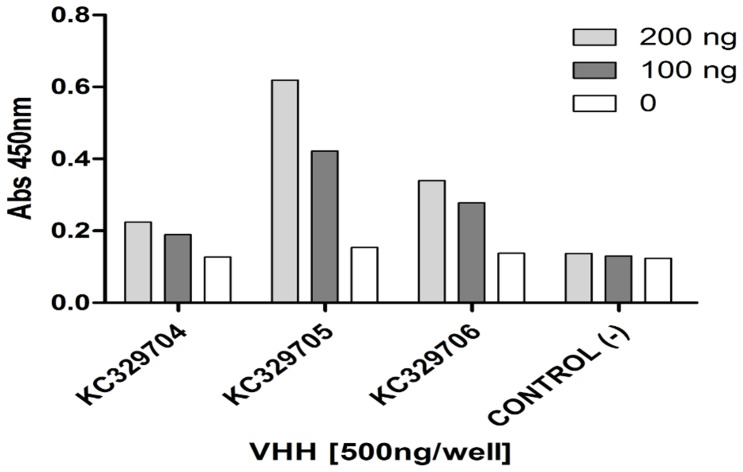
ELISA using VHH for hantavirus recombinant viral antigen detection in solution. For detection of soluble prNΔ_85_, 500 ng/well of KC329704, KC329705, and KC329706 anti-prNΔ_85_VHHs were adsorbed to a solid surface. After blocking unspecific bind sites, 0, 100, and 200 ng/well of prNΔ_85_ were added to the reaction. Subsequently, Mab 432/6BF was added and the reaction revealed ater incubation of goat anti-mouse IgG peroxidase conjugated and TMB substrate. To perform the negative control no anti-prNΔ_85_VHHs were added to the plates. The clone KC329705 was able to detect up to 100 ng of recombinant antigen in solution.

**Figure 6:**
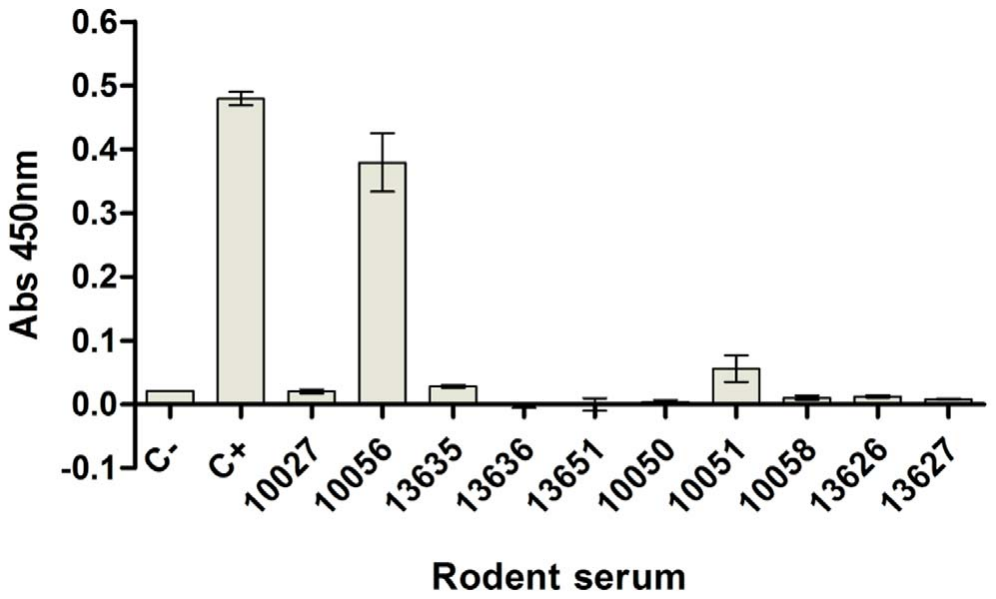
Detection of hantavirus naturally infected rodents by the assay using anti-prNΔ_85_VHH (KC329705) as captor. To detect viral antigens in rodent serum samples, about 500 ng/well of KC329705 anti-prNΔ_85_ VHH were adsorbed in microwell plates. Unspecific sites were blocked and infected rodent sera, diluted 1∶100 in blocking buffer, were added to the wells. After washing, the Mab 432/6BF was used as tracers. The wells were washed and the goat anti-mouse IgG peroxidase conjugated incubated. During the detection of native viral antigen, the hantavirus recombinant nucleoprotein (200 ng/well) was used as a positive control. While the negative control was performed adding no anti-prNΔ_85_VHHs to the plates.

To determine whether the anti-prNΔ_85_VHHs could be used as a diagnostic tool it was ensured that the recombinant hantavirus nucleoprotein in solution could be detected by ELISA. The KC329705 VHH was able to better recognize the prNΔ_85_ than KC329704 and KC329706 VHHs, detecting up to 100 ng of recombinant antigen in solution (Figure 5). Using rodent samples of hantavirus naturally infected animals to check VHH's ability to detect authentic hantaviral samples, the results demonstrated that the KC329705 VHH was able to detect one out of five positive samples (Figure 6).

## Discussion

Due to their recognition ability, and affinity and specificity for a wide diversity of antigens, antibodies are nowadays essential tools for biomedical research, diagnosis and treatment of diseases [41]. The search for functional antibody fragments that maintains or improves their essential properties, such as physicochemical characteristics, pharmacokinetic properties and low immunogenicity is always increasing. Thus, particular characteristics presented by the single domains (VHH) of camelid heavy chain antibodies have stimulated researchers to develop studies aiming to use these fragments as components of diagnostic devices or in anti-viral therapy [9], [11], [12], [42]–[46].

Considering that the target nucleoprotein N, in addition to having a high immunogenicity, presents conserved antigenic sites among several serotypes and genotypes of hantavirus [47] and participates in the virus replication process [32], anti-prNΔ_85_ VHHs could be appropriate for the development of prototypes for the rapid diagnosis of hantavirus infections in human and rodent samples, as well as for intrabodies used for monitoring and viral inhibition during the early course of infection.

In order to obtain specific anti-prNΔ_85_ VHHs, an immune library was constructed after the immunization of one *Lama glama* with the recombinant N protein, prNΔ_85_. The immunization scheme, based on protocol described by Chotwiwatthanakun *et al*. [48], with modifications in terms of time interval between the injections, total number of injection sites, and injection volumes, seemed to be very effective. Comparable to Richard et al. [49], we perceived a decrease in the animal humoral response to prNHΔ_85_ after the second boost (day 28). Thus, a third injection was performed. The llama immune response increased again and the final animal bleed showed an antiserum titre higher than that observed by previous studies [49]. With a titer of 3×10^7^ individual clones, our immune library aimed besides ensuring the affinity maturation of antibodies *in vivo*, allow for the selection of VHHs able to recognize a wide diversity of epitopes with affinity in a nano or even in a picomolar range [9], [50]. As described by Russel and Model [51], after two rounds of selection, specific antibodies to the target antigen could be identified by ELISA [52]. As expected, 50% of randomly selected clones were able to recognize the protein prNΔ_85_ by ELISA after the first round of selection. This percentage increased to 73% after the second round, pointing to a possible increase in clonal affinity and specificity. Furthermore, all selected VHHs were not capable of interacting with yellow fever attenuated virus, indicating that the selected VHHs could be specific for hantavirus.

Even though a large sequence variety was expected, only 11 different profiles were identified. Of these, four sequence patterns predominated, suggesting that the selected VHHs originated from four different B-cell lineages. It is important to note that the structural difference between the human VH and the camelid VHH is located mainly in the CDR regions [53]. Based mainly on CDR homology, sequences that shared high identity were clustered together. The notable feature of VHH, the relatively long CDR3 loop, with 17 amino acids, was visualized only in the cluster III. In contrast, clusters I, II, and IV presented a CDR3 profile similar to the average 12.7 amino acids in the human heavy chain CDR3 [14], [53], [54]. Moreover, the sequences presented the two cysteine residues (Cys23 and Cys94), conserved in the classical VH, however no additional cysteines, noted in dromedary and sporadically in llama VHHs, was observed [4]. This enables the formation of a disulfide bridge between CDR1 and CDR3, stabilizing the longer CDR3 loop of VHHs [5], [53]. As described in previous studies, the well characterized VHH hallmark present in the FR2 region was identified in all sequences. These amino acid substitutions distinguish VHH from the VH domains of conventional antibodies by replacing, highly conserved hydrophobic amino acids of VH domains (Val37, Gly44, Leu45, Trp47) with smaller and/or hydrophilic amino acids, reshaping the VL side of the VHH domain [52], [53].

After purification, Western blot analysis showed that all the selected anti-prNΔ_85_ VHHs, as well as the monoclonal mouse antibody, bind to the prNΔ_85_ band measuring approximately 48 kDa. This specificity for the 48 kDa band is consistent with studies which demonstrated the reactivity of mAbs with the ARAUV nucleoprotein using infected Vero E6 extracts [27].

An SPR assay was used to evaluate the association and dissociation properties between the selected VHHs and prNΔ_85_ protein. The sensograms, illustrating the kinetic profiles of the interaction among VHHs against the immobilized prNΔ_85_ protein, exhibit similar association and dissociation profiles. However the VHH KC329706 showed the best ability to recognize the ligand with good stability and bonding strength. Although presented affinity constants with similar values, the clones KC329704, KC329705 and KC329707 demonstrated lower affinity, but still with good ability to recognize the ligand. Regarding models of antigen-antibody interaction, these results are in accordance with the literature, which show that most antibodies have K_D_ values in the low micromolar (10^−6^) to nanomolar (10^−7^ to 10^−9^) range. In this study, the constants values obtained are very similar to other antigen-antibody interaction models. The SPR results presented good values of statistical standards [55], [56], [57].

The production of several recombinant N proteins from different hantavirus strains (Hantaan, Seoul, Puumala, Araraquara, Araucaria) used in the diagnosis of HFRS or HPS has been previously reported [24], [58], [59], [60]. Indirect ELISA could detect, with high sensitivity and specificity, IgM and IgG antibodies [24]. Moreover, capture EIA, developed using mAbs against the hantavirus nucleoprotein (Hantaan and Araucaria strain), showed a higher sensitivity when compared with direct EIA, especially in the early course of hantavirus infection [26], [27] [61]. Our results show that the ELISA with the KC329705 anti-prNΔ_85_VHH is able to identify the recombinant hantavirus nucleoprotein in solution in a dose-dependent manner. Additionally, it also recognized the native viral antigen in a hantavirus naturally infected rodent serum sample (in two independent experiments), indicating that it can be used for diagnostic purposes. Another fact that provides support for this observation, was seen in the SPR studies, since that clone presented a high affinity to the prNΔ_85_. It probably could not detect the virus in the other four positive samples, due to differences in viral load or limit detection issues (Table 3).

**Table 3:** Panel of samples of naturally infected rodents with hantavirus and negative controls tested with the anti-prNΔ_85_VHH (KC329705).

Sample	Rodent Specie	Sex	ELISA IgG	PCR	Hantavirus genotype	Viral Isolation	ELISA VHH
10027	*Akodon montensis*	M	Positive	Positive	Jaborá	Negative	Negative
10056	*Oxymycterus gr. Judex*	M	Positive	Positive	Araucária	Negative	**Positive**
13635	*Akodon montensis*	M	Positive	Positive	Jaborá	Negative	Negative
13636	*Akodon paranaensis*	F	Positive	Positive	Araucária	Negative	Negative
13651	*Akodon montensis*	M	Positive	Positive	Jaborá	Negative	Negative
10050	*Thaptomys nigrita*	M	Negative	ND*	ND	ND	Negative
10051	*Oryzomys angouya*	F	Negative	ND	ND	ND	Negative
10058	*Oligoryzomys nigripes*	F	Negative	ND	ND	ND	Negative
13626	*Oxymycterus sp*.	F	Negative	ND	ND	ND	Negative
13627	*Oxymycterus sp.*	F	Negative	ND	ND	ND	Negative

*ND: Not done.

In the present study, VHH was preferred over mAbs. VHHs are smaller, can be produced efficiently and cost effectively in microorganisms, can be used to inhibit viral activity and often display high stability over long periods of exposure to ambient temperature. Taken together, the selected and characterized VHHs could be used as biological input for the diagnosis of hantavirus infection through various methods, such as capture EIA or SPR. VHHs would also be suitable to use in immunohistochemistry tests or to confirm results obtained by molecular techniques or immunoenzymatic assays. Furthermore, and no less important, we speculate that anti-prNΔ_85_ VHHs could be used as an intrabody and serve as an alternative for viral activity inhibition. As demonstrated by Jiandong and coworkers [32], the blockade of the intracellular trafficking of hantavirus N protein reduces viral replication substantially [32]. Considering the high mortality associated with HPS and the need for highly accurate early diagnosis, VHH antibody fragments seems like an interesting alternative for detecting viral infections, as well as for therapeutic applications aiming to reduce injuries caused by hantaviruses.

## Supporting Information

Figure S1 **Immunoenzymatic assay of selected VHH against the hantavirus nucleoprotein.** A: Positive VHHs derived from the first round of selection. B and C: anti-prNΔ_85_ VHHs selected after second the round of biopanning (a =  clone KC329699; b =  clone KC329706; c =  clone KC329700; d =  clone KC329705; e =  clone KC3297003; f =  KC329707; g =  clone KC329708; h =  clone KC329704; i =  clone KC329701; j =  clone KC329702; k =  clone KC329698). All measurements were performed in triplicate. Cut off point: 3 mean OD of the samples in the negative wells samples plus 3 standard deviations. Llama imune serum was used as a positive control. The negative control was performed using the llama pre-immune serum.
(TIF)

Figure S2 **Immunoenzymatic assay of selected VHH against the yellow fever virus.** Positive VHHs derived from the first (A) and second (B) rounds of selection were not able to react with yellow fever attenuated virus. All measurements were performed in triplicate. Cut off point: 3 mean OD of the samples in the negative wells samples plus 3 standard deviations. Llama imune serum was used as a positive control. The negative control was performed using the llama pre-immune serum.
(TIF)
