# Supplementary figures and images for: Novel Camelid Antibody Fragments Targeting Recombinant Nucleoprotein of Araucaria hantavirus: A Prototype for an Early Diagnosis of Hantavirus Pulmonary Syndrome
Source: PLoS One. 2014 Sep 22;9(9):e108067. doi: 10.1371/journal.pone.0108067 (PMC4171512; doi:10.1371/journal.pone.0108067)

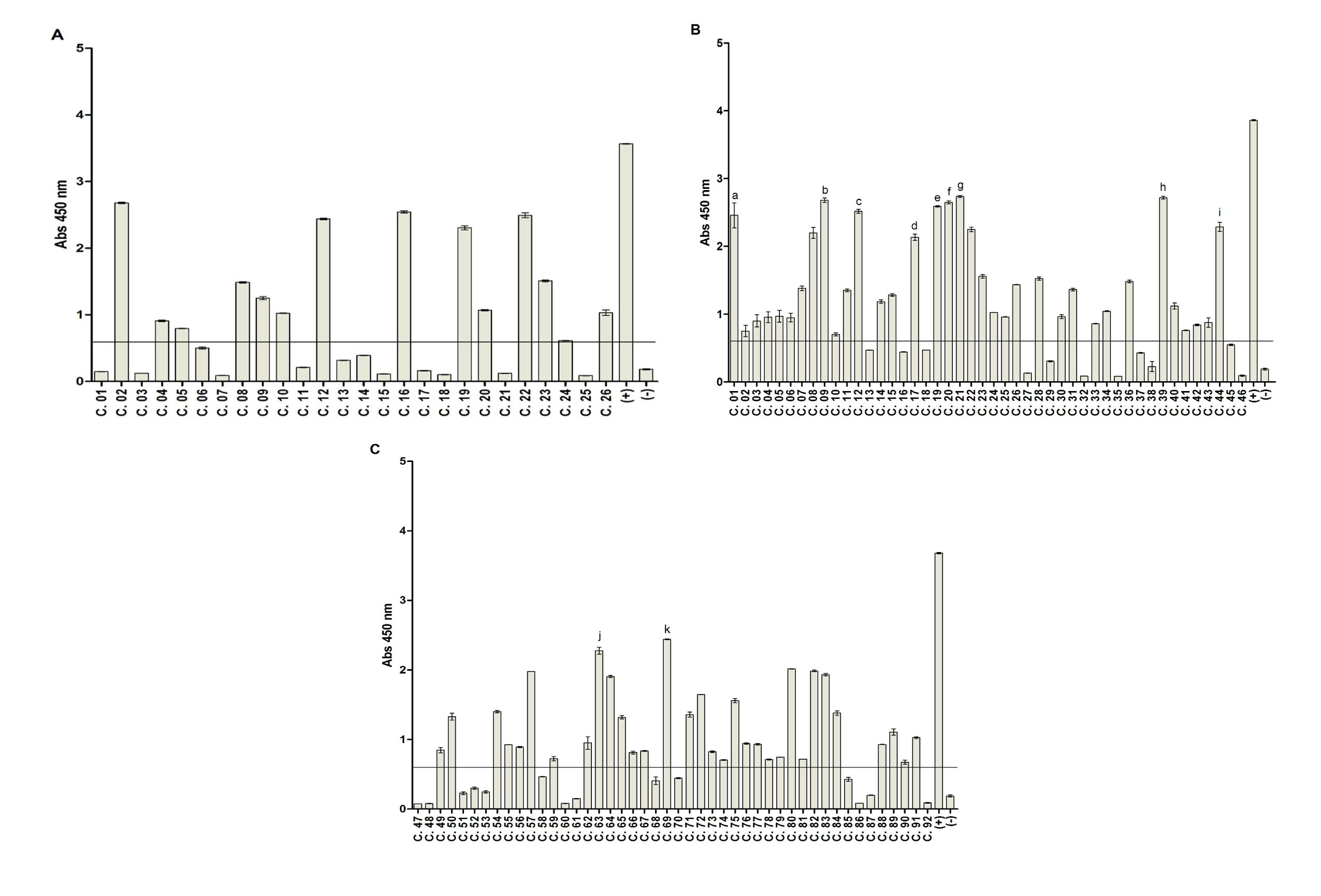

Supplement: Figure S1 — Immunoenzymatic assay of selected VHH against the hantavirus nucleoprotein. A: Positive VHHs derived from the first round of selection. B and C: anti-prNΔ85 VHHs selected after second the round of biopanning (a = clone KC329699; b = clone KC329706; c = clone KC329700; d = clone KC329705; e = clone KC3297003; f = KC329707; g = clone KC329708; h = clone KC329704; i = clone KC329701; j = clone KC329702; k = clone KC329698). All measurements were performed in triplicate. Cut off point: 3 mean OD of the samples in the negative wells samples plus 3 standard deviations. Llama imune serum was used as a positive control. The negative control was performed using the llama pre-immune serum. (TIF) [file pone.0108067.s001.tif]

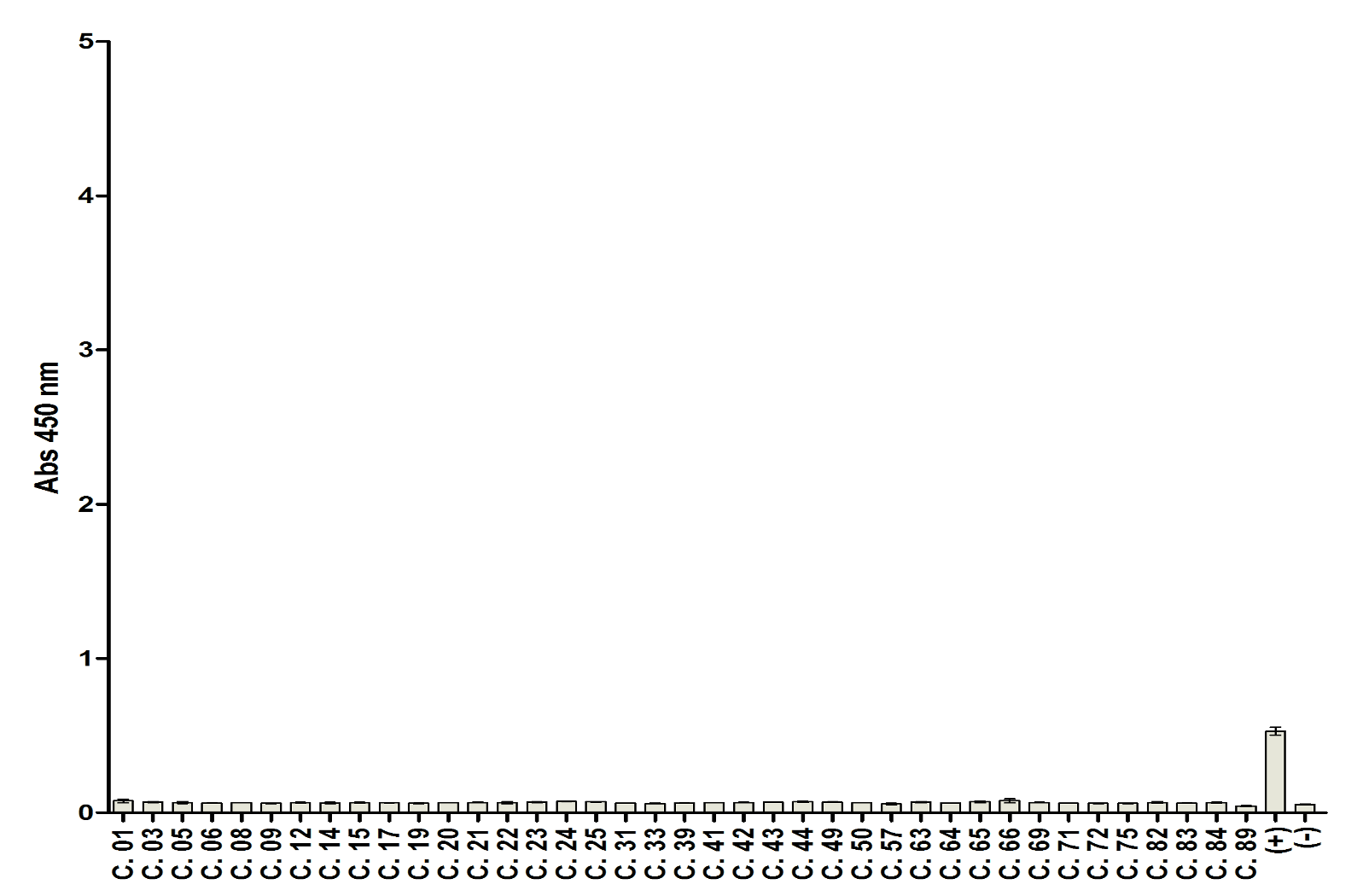

Supplement: Figure S2 — Immunoenzymatic assay of selected VHH against the yellow fever virus. Positive VHHs derived from the first (A) and second (B) rounds of selection were not able to react with yellow fever attenuated virus. All measurements were performed in triplicate. Cut off point: 3 mean OD of the samples in the negative wells samples plus 3 standard deviations. Llama imune serum was used as a positive control. The negative control was performed using the llama pre-immune serum. (TIF) [file pone.0108067.s002.tif]
